# ABA crosstalk with ethylene and nitric oxide in seed dormancy and germination

**DOI:** 10.3389/fpls.2013.00063

**Published:** 2013-03-26

**Authors:** Erwann Arc, Julien Sechet, Françoise Corbineau, Loïc Rajjou, Annie Marion-Poll

**Affiliations:** ^1^Institut Jean-Pierre Bourgin (UMR1318 INRA – AgroParisTech), Institut National de la Recherche Agronomique, Saclay Plant ScienceVersailles, France; ^2^UFR de Physiologie végétale, AgroParisTechParis, France; ^3^Germination et Dormance des Semences, UR5 UPMC-EAC 7180 CNRS, Université Pierre et Marie Curie-Paris 6Paris, France

**Keywords:** abscisic acid, dormancy, ethylene, germination, hormone, nitric oxide, seed

## Abstract

Dormancy is an adaptive trait that enables seed germination to coincide with favorable environmental conditions. It has been clearly demonstrated that dormancy is induced by abscisic acid (ABA) during seed development on the mother plant. After seed dispersal, germination is preceded by a decline in ABA in imbibed seeds, which results from ABA catabolism through 8′-hydroxylation. The hormonal balance between ABA and gibberellins (GAs) has been shown to act as an integrator of environmental cues to maintain dormancy or activate germination. The interplay of ABA with other endogenous signals is however less documented. In numerous species, ethylene counteracts ABA signaling pathways and induces germination. In Brassicaceae seeds, ethylene prevents the inhibitory effects of ABA on endosperm cap weakening, thereby facilitating endosperm rupture and radicle emergence. Moreover, enhanced seed dormancy in *Arabidopsis* ethylene-insensitive mutants results from greater ABA sensitivity. Conversely, ABA limits ethylene action by down-regulating its biosynthesis. Nitric oxide (NO) has been proposed as a common actor in the ABA and ethylene crosstalk in seed. Indeed, convergent evidence indicates that NO is produced rapidly after seed imbibition and promotes germination by inducing the expression of the ABA 8′-hydroxylase gene, *CYP707A2*, and stimulating ethylene production. The role of NO and other nitrogen-containing compounds, such as nitrate, in seed dormancy breakage and germination stimulation has been reported in several species. This review will describe our current knowledge of ABA crosstalk with ethylene and NO, both volatile compounds that have been shown to counteract ABA action in seeds and to improve dormancy release and germination.

## INTRODUCTION

Survival of plant species mainly relies on the sexual reproduction to give birth to new individuals. In flowering plants, the seed is the main unit of dispersal and allows colonization of new geographic areas. As a consequence of the double fertilization process, a mature angiosperm seed contains a diploid embryo and protective layers comprising the triploid endosperm, a nourishing tissue for the embryo, and the seed coat of maternal origin. During development on the mother plant, after embryogenesis completion, reserve accumulation takes place and is followed, in so-called orthodox seeds, by an intense dehydration leading to low seed water content upon dispersal. In many species, a dormant state is also induced during the maturation phase, preventing pre-harvest germination and allowing seed survival until environmental conditions become suitable for germination and seedling establishment (Bentsink and Koornneef, 2008; Finkelstein et al., 2008; North et al., 2010).

Dormancy has been defined as a developmental state in which a viable seed fails to germinate under favorable environmental conditions (Bewley, 1997), but different definitions and classifications have been proposed. Finch-Savage and Leubner-Metzger (2006) summarized a classification proposed by Baskin and Baskin (2004), based on the fact that dormancy results from physiological and developmental (or morphological) properties of the seed. Dormancy is therefore divided in five classes: (1) physiological dormancy (PD) can be released by different stratification (moist chilling) treatments depending on its depth, (2) morphological dormancy (MD) is due to a delay of embryo development, (3) morphophysiological dormancy (MPD) is combining both PD and MD, (4) physical dormancy (PY) is correlated with seed coat impermeability to water and needs disruption of the seed coat (scarification) to be released, and finally (5) combinational dormancy combining PY and PD. Most species display a non-deep PD corresponding to a dormancy that can be released, depending on the species, by gibberellin (GA) treatment, stratification, scarification, or a period of dry storage (after-ripening). In this case, seeds generally combine a coat-imposed dormancy due to the covering layers of the seed (seed coat and endosperm) that prevent the radicle protrusion, and an embryo dormancy due to its incapacity to induce radicle growth.

When dormancy is released, seeds can germinate under favorable conditions, specific to each species. The germination process, that begins with seed imbibition and finishes with a developed plantlet, is divided in three distinct phases of water uptake. Phase I starts with a fast water uptake and the activation of respiratory metabolism and transcriptional and translational activities. During phase II water uptake ceases, seed reserve mobilization begins and testa rupture occurs. Later, in the third phase water uptake resumes and endosperm rupture allows radicle protrusion; then starts the post-germination phase with high water uptake, mobilization of the major part of reserves and first cell divisions, until the complete seedling development (Bewley, 1997; Nonogaki et al., 2010; Weitbrecht et al., 2011). Germination *sensu stricto* ends with radicle protrusion. It is often described has the resulting consequence of the growth potential of the embryo and the resistance of the surrounding layers. Endosperm weakening is an essential part of the modification of seed envelopes for the progress of germination and involves the activation of cell-wall modifying enzymes (Finch-Savage and Leubner-Metzger, 2006; Endo et al., 2012; Linkies and Leubner-Metzger, 2012). After dormancy release, storage/imbibition of non-dormant seeds in unfavorable conditions for germination can trigger a secondary dormancy. This is a way to protect seeds against germination too late in the year and induce a seasonal cycling of dormancy level in seeds (Cadman et al., 2006; Footitt et al., 2011).

The regulation of seed dormancy and germination by the hormonal balance between abscisic acid (ABA) and GA, in response to environmental signals, is well documented in a number of recent reviews (Finkelstein et al., 2008; Seo et al., 2009; Nambara et al., 2010; Nonogaki et al., 2010; Weitbrecht et al., 2011; Graeber et al., 2012; Rajjou et al., 2012). The present review will describe recent knowledge about key players in the ABA metabolism and signaling pathways that control dormancy induction and maintenance and convergent evidences supporting the role of two other signaling compounds, nitric oxide (NO) and ethylene, in dormancy breakage and germination, and their interactions with ABA metabolism and signaling pathways.

## ABA HOMEOSTASIS AND SIGNALING IN DORMANCY CONTROL

### ABA SYNTHESIS

**FIGURE 1:**
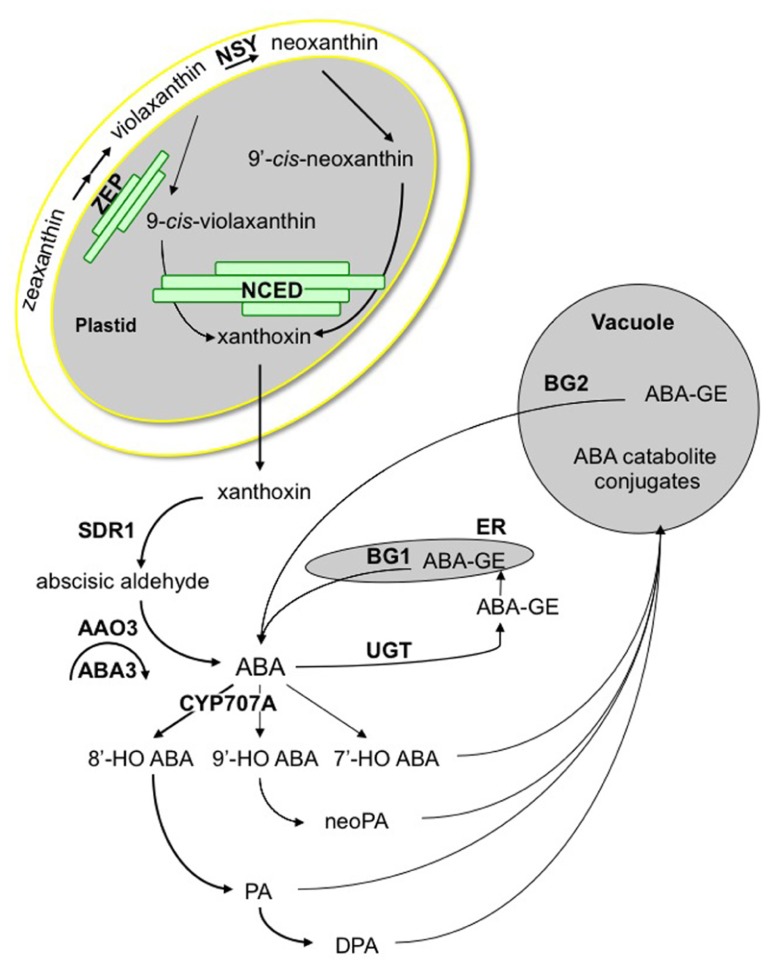
** ABA metabolism pathway.** Zeaxanthin conversion into violaxanthin is catalyzed by zeaxanthin epoxidase (ZEP). ABA4 is involved in the synthesis of neoxanthin, which is then *cis*-isomerized, together with violaxanthin, by an unknown isomerase. Carotenoid cleavage is catalyzed by a family of 9-*cis*-epoxycarotenoid dioxygenases (NCED) to form xanthoxin. Xanthoxin moves to the cytosol by an unknown mechanism and is converted into abscisic aldehyde by a short-chain dehydrogenase reductase (SDR1), which is then oxidized into ABA by an abscisic aldehyde oxidase (AAO3). Sulfuration of AAO3 molybdenum co-factor by ABA3 is necessary for enzyme activity. The 8′-hydroxylation by CYP707A enzymes is thought to be the predominant pathway for ABA catabolism. Hydroxy-groups of ABA and its catabolites, phaseic acid (PA), neoPA, and dihydrophaseic acid (DPA) are targets for conjugation. ABA-glucose ester is formed by ABA glucosyltransferases (UGT) and hydrolyzed by glucosidases, including BG1 and BG2.

Abscisic acid is formed by cleavage of C_40_ oxygenated carotenoids, also called xanthophylls, which are produced in plastids from C_5_ precursors (Ruiz-Sola and Rodrïguez-Concepción, 2012). Key genes encoding enzymes of the ABA biosynthesis pathway have been identified through mutant selection for altered germination phenotypes, giving further evidence of the major role of ABA in the regulation of seed dormancy and germination (**Figure 1**). For instance, the first ABA-deficient mutant, identified in* Arabidopsis thaliana*, was isolated in a GA biosynthesis mutant *ga1* suppressor screen, on its ability to germinate in the absence of GA. It was shown to be defective in zeaxanthin epoxidase (ZEP) activity, like a *Nicotiana plumbaginifolia* mutant selected later on its early germination phenotype (Koornneef et al., 1982; Marin et al., 1996). ZEP catalyzes the epoxidation of zeaxanthin into violaxanthin and is encoded, in *Arabidopsis*, by the *ABA1* gene (Audran et al., 2001; Xiong et al., 2002). Violaxanthin is then converted into neoxanthin, by neoxanthin synthase (NSY), likely encoded by the *Arabidopsis* *ABA4* gene (Dall’Osto et al., 2007; North et al., 2007). Despite impairment in *ABA4* function completely prevents neoxanthin synthesis, the *aba4* mutant exhibits no obvious dormancy phenotype, due the formation of *cis*-violaxanthin by an alternate pathway (North et al., 2007). Both *cis*-violaxanthin and *cis*-neoxanthin cleavage gives rise to xanthoxin, the C_15_ aldehyde precursor of ABA. Since *cis*-isomerization of violaxanthin and neoxanthin is required prior to cleavage, an unknown isomerase might be involved. The *VIVIPAROUS14* (*VP14*) gene in maize (*Zea mays*) has been shown to encode a 9-*cis*-epoxycarotenoid dioxygenase (NCED), which catalyzes the oxidative cleavage of either 9′-*cis*-neoxanthin or 9-*cis*-violaxanthin (Schwartz et al., 1997; Tan et al., 1997). *NCED* genes have been then identified in a number of other plant species (Nambara and Marion-Poll, 2005). In *Arabidopsis*, *VP14*-related gene family is composed of nine members, five of which (*NCED2*, *NCED3*, *NCED5*, *NCED6*, and *NCED9*) encode xanthoxin-producing enzymes (Iuchi et al., 2001; Toh et al., 2008).

In *Arabidopsis* plastids, ZEP is associated mainly to envelope and slightly to thylakoid membranes (**Figure 1**). In contrast NSY/ABA4 is presumably tightly bound to the envelope since this protein is predicted to contain four transmembrane domains and is exclusively found in the envelope fraction (Joyard et al., 2009). In contrast, NCED proteins have been detected either in stroma or thylakoid membrane-bound compartments, or both (Tan et al., 2003). In addition, recent VP14 structural analysis suggested that this enzyme might penetrate the surface of thylakoid membrane to access and transfer carotenoid substrates to its catalytic center (Messing et al., 2010). The scattered location of ZEP, NSY, and NCED suggests that the production of xanthoxin inside plastids may require transport mechanisms of lipid-soluble carotenoid molecules, which are not currently understood. Since the following enzymatic reactions take place in the cytosol, xanthoxin is also presumed to migrate from plastid to cytosol by a still unknown mechanism.

Abscisic aldehyde is synthesized from xanthoxin, by an enzyme belonging to short-chain dehydrogenase/reductase family, which is named SDR1 and is encoded by the *ABA2* gene in *Arabidopsis* (Rook et al., 2001; Cheng et al., 2002; Gonzalez-Guzman et al., 2002). The oxidation of the ABA-aldehyde is the final step of ABA biosynthesis, and is catalyzed by an abscisic aldehyde oxidase. In *Arabidopsis*, four homologous aldehyde oxidase (*AAO*) genes have been characterized, but only one of them, *AAO3*, encodes a protein that has proven activity on abscisic aldehyde (Seo et al., 2000). Activity of this molybdoenzyme requires the activation of its molybdenum co-factor (Moco) by addition of a sulfur atom to the Mo center, which is catalyzed by a Moco sulfurase, which has been named ABA3 in *Arabidopsis* (Bittner et al., 2001; Xiong et al., 2001).

### ABA CATABOLISM

Abscisic acid inactivation is a crucial mechanism to fine-tune ABA levels, which occurs by either oxidation or conjugation (**Figure 1**). The major catabolic route is the 8′-hydroxylation of ABA by the CYP707A subfamily of P450 monooxygenases (Kushiro et al., 2004; Saito et al., 2004). Spontaneous 8′-hydroxy-ABA isomerization gives rise to phaseic acid (PA), which is then converted to dihydrophaseic acid (DPA) by a still unknown reductase. ABA can also be hydroxylated at the C-7′ and C-9′ positions. As 8′-hydroxylation, 9′-hydroxylation is catalyzed by CYP707A as a side reaction, and neoPA is then formed by spontaneous isomerization (Zhou et al., 2004; Okamoto et al., 2011). The conjugation of ABA with glucose to form the ABA-glucose ester (ABA-GE) is catalyzed by an ABA glucosyltransferase, and in *Arabidopsis* only UGT71B6 exhibits a selective glucosylation activity toward the natural enantiomer (+)-ABA (Lim et al., 2005; Priest et al., 2006). Subsequent hydrolysis of conjugates constitutes an alternative pathway for ABA synthesis in response to dehydration stress. Two glucosidases BG1 and BG2, localizing respectively in the endoplasmic reticulum or the vacuole, have been identified (Lee et al., 2006; Xu et al., 2012).

Deficiency in either ABA synthesis or ABA inactivation by 8′-hydroxylation leads to strong dormancy phenotypes, respectively dormancy loss or strengthening (Nambara and Marion-Poll, 2005; Seo et al., 2009; Nambara et al., 2010). In contrast, reports on functional analysis of mutant or overexpressing lines in ABA conjugation or ABA-GE hydrolysis did not yet describe the implication of these processes in dormancy control. Nevertheless, ABA conjugation may contribute to ABA breakdown upon germination, as shown in lettuce (*Lactuca sativa*; Chiwocha et al., 2003).

### ABA SIGNALING PATHWAY

**FIGURE 2:**
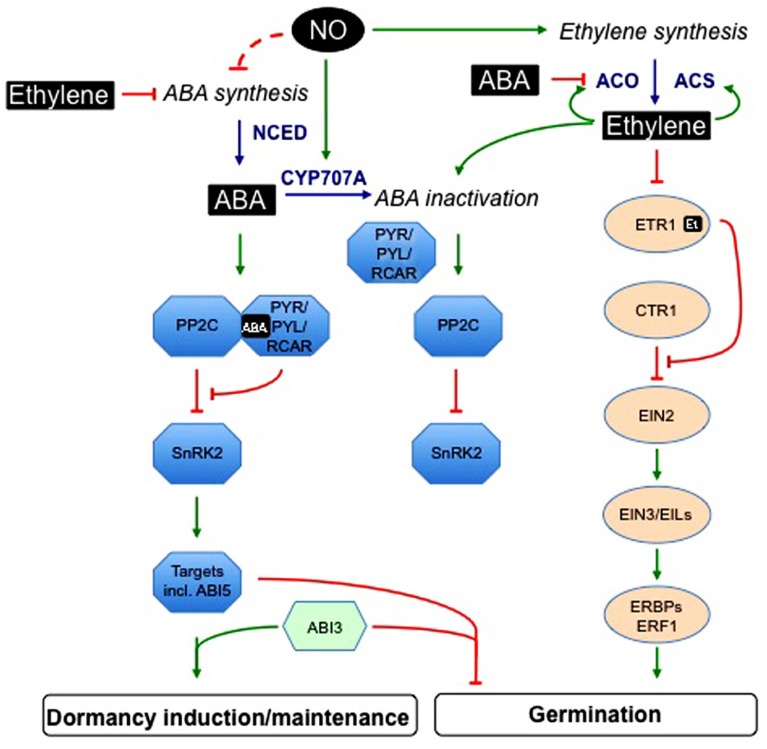
**Interactions between ethylene, abscisic acid, and nitric oxide signaling pathways in the regulation of seed germination and dormancy**. This scheme is based on genetic analyses, microarray data, and physiological studies on seed responsiveness to ABA, ethylene, or NO. ABA binding to PYR/PYL/RCAR receptor induces the formation of a protein complex with PP2C and the inhibition of phosphatase activity. In the absence of ABA, PP2C dephosphorylate SnRK2. When ABA is present, PP2C binding to the receptor releases inhibition of SnRK2 activity, which can phosphorylate downstream targets, including ABI5-related transcription factors. Interactions between ABI3 and ABI5 mediate transcriptional regulation of ABA-responsive genes. Ethylene positively regulates its own biosynthesis, by acting on ACC synthesis catalyzed by ACS and subsequent conversion to ethylene by ACO. This last step is also subject to ABA inhibition. Ethylene is perceived by receptors (among which ETR1) located in the endoplasmic reticulum; its binding leads to the deactivation of the receptors that become enable to recruit CTR1. Release of CTR1 inhibition allows EIN2 to act as a positive regulator of ethylene signaling pathway. EIN2 acts upstream of nuclear transcription factors, such as EIN3, EILs, and ERBPs/ERFs. Ethylene down-regulates ABA accumulation by both inhibiting its synthesis and promoting its inactivation, and also negatively regulates ABA signaling. In germinating seeds, NO enhances ABA catabolism and may also negatively regulate ABA synthesis and perception. Moreover, NO promotes both ethylene synthesis and signaling pathway. ABA, abscisic acid; ABI3, ABA insensitive3; ABI5, ABA insensitive5; ACC, 1-aminocyclopropane 1-carboxylic acid; ACO, ACC oxidase; ACS, ACC synthase; CTR1, constitutive triple response 1; CYP707A, ABA-8′-hydroxylase; EIL, EIN3-like; EIN, ethylene-insensitive; EREBP, ethylene-responsive element binding protein; ERF, ethylene response factor; Et, ethylene; ETR1, ethylene receptor1; NCED, 9-cis-epoxycarotenoid dioxygenase; NO, nitric oxide; PP2C, clade A type 2C protein phosphatases; PYR/PYL/RCAR, pyrabactin resistance1/PYR1-like/regulatory components of ABA receptor; SnRK2, group III sucrose non-fermenting-1-related protein kinase 2; a dashed line is used when regulatory targets are not precisely identified.

Genetic analyses suggest that PYR/PYL/RCAR (pyrabactin resistance1/PYR1-like/regulatory components of ABA receptor) ABA receptors, clade A type 2C protein phosphatases (PP2C) and group III sucrose non-fermenting1-related protein kinase2 (SnRK2) subfamily are essential core components of the upstream signal transduction network that regulates ABA-responsive processes, including dormancy and germination (reviewed in Cutler et al., 2010). PYR/PYL/RCAR proteins constitute a 14-member family, belonging to the START-domain superfamily, also called Bet v I-fold (Ma et al., 2009; Park et al., 2009). ABA binding induces receptor conformation changes allowing the formation of a protein complex with PP2C and the inhibition of phosphatase activity (**Figure 2**). The clade A PP2C, including ABA INSENSITIVE1 (ABI1) and ABI2, also interact with three SnRK2 (SnRK2.2, SnRK2.3, and SnRK2.6) and, in the absence of ABA, dephosphorylate a serine residue whose phosphorylation is required for kinase activity (Soon et al., 2012). When ABA is present, PP2C binding to the receptor releases inhibition of SnRK2 activity, which can phosphorylate downstream targets.

Other types of receptors and a large number of genes, whose mutations alter ABA germination sensitivity, have been reported to participate in ABA signaling. In particular, regulatory mechanisms such as RNA processing, RNA/protein stability or chromatin remodeling have an important role. However, they will not be detailed here, since their role in ABA crosstalk with ethylene and NO in seeds still requires further investigation. In *Arabidopsis* seeds, extensive evidence including mutant phenotypes strongly supports a central role of the PYR/PYL, PP2C, SnRK2 complex in ABA signaling (reviewed in Cutler et al., 2010; Nambara et al., 2010). Germination of a *pyr/pyl* sextuple mutant is highly insensitive to ABA, as also observed for the *snrk2.2 snrk2.3 snrk2.6* triple mutant (Fujii and Zhu, 2009; Gonzalez-Guzman et al., 2012). Moreover the *snrk2* triple mutant exhibits loss of dormancy and even seed vivipary under high humidity conditions (Nakashima et al., 2009). Conversely, in accordance with PP2C being negative regulators of ABA signaling, germination in triple *pp2c* mutants was slower than in wild type and was inhibited by very low ABA concentrations (Rubio et al., 2009). In contrast, the gain-of-function mutations *abi1-1* and *abi2-1*, which prevent PP2C binding to PYL/PYR/RCAR, lead to ABA insensitivity and reduced dormancy (Ma et al., 2009; Park et al., 2009).

Basic leucine zipper transcription (bZIP) factors of the ABA-RESPONSIVE ELEMENTS (ABRE) BINDING FACTOR/ABA RESPONSE ELEMENT BINDING FACTOR/ABA INSENSITIVE5 (ABF/AREB/ABI5) clade have been shown in different species to constitute SnRK2 downstream targets and regulate ABRE containing genes (Johnson et al., 2002; Kobayashi et al., 2005; Umezawa et al., 2009). Several family members are expressed at different seed stages and exhibit partially redundant or antagonistic functions, and ABI5 appears to have a predominant role in the regulation of a subset of late embryogenesis abundant (LEA) proteins during late seed development (Bensmihen et al., 2002; Finkelstein et al., 2005). *abi5* mutation confers ABA-insensitive germination, but it does not impair seed dormancy, suggesting that other factors might be involved in dormancy induction (Finkelstein, 1994). Nevertheless, ABI5 has been clearly proven to act as a major inhibitor of germination processes in imbibed seeds, notably through its up-regulation by stress-induced ABA accumulation (Lopez-Molina et al., 2001; Piskurewicz et al., 2008). ABI3/VIVIPAROUS1 (VP1) interacts with ABI5 for the regulation of a number of ABA-responsive genes during seed maturation and germination (Lopez-Molina et al., 2002; Piskurewicz et al., 2008, 2009). However, in contrast to *abi5*, *abi3* mutants do not only exhibit ABA-resistant germination, but also other phenotypes including desiccation intolerance and precocious germination. They share these maturation defects with *fusca3* (*fus3*) and *leafy cotyledon2* (*lec2*) mutants, which, like *abi3*, carry mutations in B3 transcription factor family genes. These factors form a complex network regulating the expression of reserve storage and LEA genes by their binding to RY motif, and it has been suggested that the lack of dormancy induction in mutants might indirectly result from early seed developmental defects (Gutierrez et al., 2007; Finkelstein et al., 2008; Santos-Mendoza et al., 2008; Graeber et al., 2012). Nevertheless *fus3* mutation has been shown to affect ABA levels in developing seeds (Gazzarrini et al., 2004). In addition, ABA-specific phenotypes of *abi3/vp1* mutants strongly suggest an involvement in ABA-regulated dormancy induction, but downstream dormancy genes still remain elusive. Nevertheless, one of these might be the recently identified *seed dormancy4* (*Sdr4*) gene in rice, which encodes a nuclear protein of unknown function (Sugimoto et al., 2010).

The *Arabidopsis* *DELAY OF GERMINATION1* (*DOG1*) gene, whose precise function is still unknown, has been identified as a major regulator of seed dormancy (Bentsink et al., 2006). In accordance, protein accumulation in dry seeds well correlates with dormancy depth, and both transcript and protein levels are increased upon cool conditions of seed maturation, which increase seed dormancy (Kendall et al., 2011; Nakabayashi et al., 2012). Despite *dog1* dormancy phenotypes are similar to ABA synthesis and signaling mutants, current evidence suggests that DOG1 and ABA act in independent pathways. Nevertheless regulation of dormancy depth by DOG1 requires a functional ABA signaling pathway (Nakabayashi et al., 2012), and DOG1 has been reported to be implicated in the ABA-mediated sugar signaling pathway, together with ABI4, an APETALA2 transcription factor involved in reserve mobilization at germination (Penfield et al., 2006; Teng et al., 2008). Another mutation, named *despierto* (*dep*), also causes dormancy loss (Barrero et al., 2010). *DEP* gene encodes a C3HC4 RING (Really Interesting New Gene)-finger protein, whose targets are unknown. In addition to similarity in mutant phenotypes, expression of both *DEP* and *DOG1* genes is maximal during late seed development and decreases during imbibition. Moreover *dep* mutation reduces *DOG1* transcript levels in developing seeds and vice versa. It also down-regulates the expression of several ABA biosynthesis and signaling genes, including *NCED6*, *NCED9*, and *ABI3*, suggesting its action in dormancy induction may involve the ABA signaling pathway (Barrero et al., 2010).

### SPATIOTEMPORAL REGULATION OF ABA LEVEL AND SIGNALING IN DORMANCY AND GERMINATION

Abscisic acid is produced in all seed tissues (testa, endosperm, embryo), as suggested by the spatiotemporal expression of ABA biosynthesis genes (Lefebvre et al., 2006; Frey et al., 2012). However, ABA accumulated in seeds also originates from synthesis in vegetative tissues and transport to the seed (Frey et al., 2004; Kanno et al., 2010). Several ABA transporters have been recently identified, which belong to either the ATP-binding cassette (ABC) or nitrate transporter 1 (NRT1)/peptide transporter (PTR) families (Kang et al., 2010; Kuromori et al., 2010; Kanno et al., 2012). ABC transporter G family member 25 (ABCG25) functions as a plasma membrane ABA exporter, whereas both ABCG40 and AIT1 (ABA IMPORTER1) are plasma membrane uptake transporters. Despite mutations in these three genes induce alterations in germination sensitivity to ABA, suggesting a possible function in seeds, the precise contribution of any of them to either ABA supply from mother plant to seeds or its translocation between maternal and/or embryonic seed tissues needs further investigation. Another ABC transporter gene, ABCG22, has been reported to be involved in ABA-regulated water stress tolerance, but its function in ABA transport remains uncertain (Kuromori et al., 2011). ABA levels are maximal during mid-seed development, with a large fraction produced in maternal tissues (Karssen et al., 1983; Kanno et al., 2010). Maternal ABA has a major contribution to the regulation of many aspects of seed development, but only ABA produced by zygotic tissues at late maturation stages imposes dormancy (Karssen et al., 1983; Frey et al., 2004).

Carotenoid cleavage by NCED and ABA inactivation by CYP707A 8′-hydroxylase have been proven to constitute key regulatory steps for the control of ABA levels, which affect seed dormancy and germination in response to environmental cues (Nambara and Marion-Poll, 2005; Seo et al., 2009; Nambara et al., 2010). Among the five *Arabidopsis* *NCED* genes, *NCED6* and *NCED9* exhibit the highest expression levels in developing seeds and show distinctive expression patterns. *NCED6* is specifically expressed in endosperm, whereas *NCED9* expression is detected in testa and embryo. Furthermore mutant analysis indicated that ABA production in both embryo and endosperm contributes to dormancy induction (Lefebvre et al., 2006; Frey et al., 2012). In barley (*Hordeum vulgare*), the two *HvNCED* genes also exhibit differential spatiotemporal patterns of expression. In contrast to *HvNCED2*, *HvNCED1* transcript levels vary depending on environmental conditions during grain development and modulate ABA accumulation at late maturation stages (Chono et al., 2006). ABA inactivation by CYP707A during seed maturation also regulates dry seed ABA levels and dormancy depth, as deduced from *cyp707a* mutant analysis (Okamoto et al., 2006). Moreover, the seed dormancy increase under cold-maturation conditions is not only correlated with *DOG1* up-regulation, as mentioned above, but also with *CYP707A2* down-regulation (Kendall et al., 2011).

Upon imbibition, dormancy maintenance and germination are also regulated by both ABA catabolism and neo-synthesis. A decrease in ABA levels at imbibition has been observed in both dormant and non-dormant seeds in several species; nevertheless dormant seeds maintain higher ABA levels and in accordance exhibit lower *CYP707A* transcript levels, as shown in *Arabidopsis* and barley (Millar et al., 2006). Barley *HvABA8’OH1* transcripts were detected in coleorhiza cells near the root apex and *Arabidopsis* *CYP707A2* in endodermis and micropylar endosperm next to the radicle (Millar et al., 2006; Okamoto et al., 2006). Moreover, it is well documented in several species that unfavorable light or temperature conditions prevent germination by coordinated regulation of *NCED* and *CYP707A* gene expression (Seo et al., 2006; Gubler et al., 2008; Toh et al., 2008; Leymarie et al., 2009; Argyris et al., 2011). Furthermore, dormancy cycling by seasonal variation of soil temperature has been recently linked to the regulation of ABA metabolism and signaling genes. Deep dormancy in winter is correlated with increased ABA levels and *NCED6* expression, together with that of *DOG1* and *MOTHER OF FLOWERING LOCUS T* (*MFT*). *MFT* encodes a phosphatidylethanolamine-binding protein, which is regulated by *ABI3* and *ABI5*, and feedback regulates ABA signaling by repressing *ABI5* (Xi et al., 2010). In contrast, shallow dormancy in summer is correlated with a reduction in ABA levels and an up-regulation of *CYP707A2* and *ABI2*, which negatively regulates ABA signaling (Footitt et al., 2011).

In *Arabidopsis*, despite endosperm consists in a single cell layer in mature seeds, convergent evidence demonstrated its major role in ABA control of seed dormancy and germination. Firstly, whereas the removal of whole seed coat (endosperm and testa) releases mechanical constraints and allows development of embryos dissected from dormant seeds, the preservation of the endosperm after testa removal maintains dormancy (Bethke et al., 2007a). Secondly, using a “seed coat bedding assay,” Lee et al. (2010) showed that diffusion of endospermic ABA from dormant seed envelopes could prevent growth of non-dormant embryos, including those of ABA-deficient *aba2* mutants. In isolated embryos, translocated ABA was able to induce ABI5 protein accumulation, whose level was correlated with dormancy maintenance. In addition, in a previous study, *ABI5* transcript was detected in the embryo and the micropylar endosperm of imbibed seeds, suggesting a role in the inhibition of both embryo growth and endosperm rupture by ABA (Penfield et al., 2006). The tissue-specificity of ABA sensitivity is also likely regulated by the spatiotemporal expression of upstream ABA signaling components, as suggested by the differential expression of PYR/PYL genes in embryo and/or endosperm of imbibed seeds (Gonzalez-Guzman et al., 2012).

## ETHYLENE BIOSYNTHESIS, SIGNALING, AND ABA CROSSTALK IN SEED GERMINATION

### ETHYLENE BIOSYNTHESIS AND SIGNALING

**FIGURE 3:**
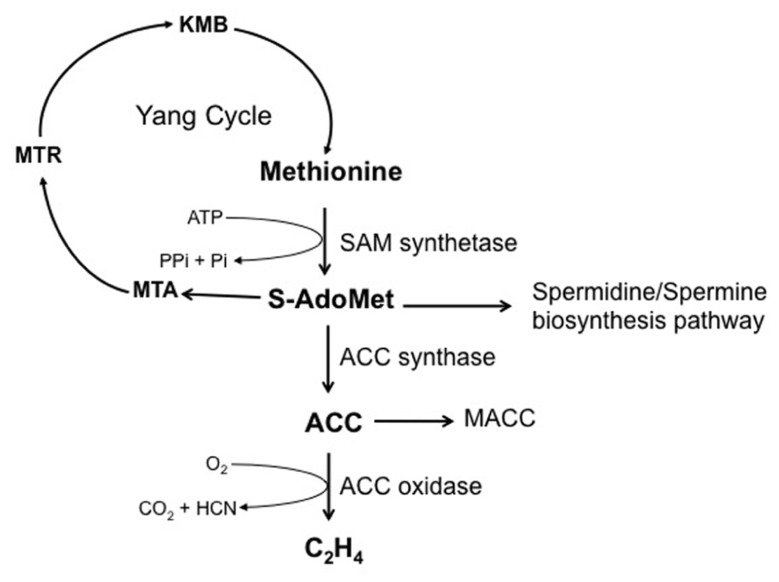
**Ethylene biosynthesis pathway**. *S*-adenosyl-methionine (*S*-AdoMet) is synthesized from the methionine by the *S*-adenosyl-methionine synthetase (SAM synthetase) with one ATP molecule expensed per *S*-AdoMet synthesized. *S*-AdoMet is then converted to 1-aminocyclopropane-1-carboxylic acid (ACC) by ACC synthase, 5′-methylthioadenosine (MTA) being a by-product. MTA is recycled to methionine by successive enzymatic reactions involving various intermediates (MTR, 5-methylthioribose; KMB, 2-keto-4-methylthiobutyrate), which constitute the methionine (Yang) cycle. *S*-AdoMet is also the precursor of the spermidine/spermine biosynthesis pathway. Ethylene production is catalyzed by the ACC oxidase using ACC as substrate, and generates carbon dioxide and hydrogen cyanide. Malonylation of ACC to malonyl-ACC (MACC) reduces ACC content and consequently ethylene production.

Ethylene biosynthesis pathway in germinating seeds is the same as that described in other plant organs (**Figure 3**), in which *S*-adenosyl-methionine (*S*-AdoMet) and 1-aminocyclopropane-1-carboxylic acid (ACC) are the main intermediates (Yang and Hoffman, 1984; Wang et al., 2002; Rzewuski and Sauter, 2008). The first step of ethylene biosynthesis is the conversion of *S*-AdoMet to ACC catalyzed by ACC synthase (*S*-adenosyl-L-methionine methylthioadenosine-lyase, ACS), the by-product being 5′-methylthioadenosine (MTA), which is recycled back to methionine through the Yang cycle (Yang and Hoffman, 1984; Kende, 1993). The second step corresponds to the oxidation of ACC by ACC oxidase (ACO) to form ethylene, CO_2_, and hydrogen cyanide (HCN). Cyanide produced during this final step of ethylene synthesis is detoxified to β-cyanoalanine by β-cyanoalanine synthase (β-CAS). Both ACS and ACO are encoded by a multigene family. In *Arabidopsis*, nine active *ACS* genes have been characterized (Yamagami et al., 2003; Wang et al., 2005; Dong et al., 2011). Most of them can be induced by cycloheximide (*ACS2*, *ACS4*, *ACS6*), wounding (*ACS2*, *ACS4*), and ethylene treatment (*ACS2*, *ACS6*; reviewed in Wang et al., 2002). In addition, *ACS6* can also be induced by cyanide (Smith and Arteca, 2000) or ozone treatment (Vahala et al., 1998). ACO activity controls *in vivo* ethylene production and has fundamental contribution during seed germination (Matilla and Matilla-Vazquez, 2008; Linkies and Leubner-Metzger, 2012).

In *Arabidopsis*, five membrane-localized receptors have been identified: ethylene resistant 1 (ETR1), ETR2, ethylene response sensor 1 (ERS1), ERS2, and ethylene insensitive 4 (EIN4; **Figure 2**). Among them, ETR1 and ERS1 contain three transmembrane domains in the N-terminus and a histidine kinase domain in the C-terminus. In contrast, ETR2, EIN4, and ERS2 have four transmembrane regions and a serine–threonine kinase domain in the C-terminus (Kendrick and Chang, 2008). Binding of C_2_H_4_ to the receptors occurs in the hydrophobic N-terminal part of the receptor dimer and requires a copper co-factor (Hall et al., 2007). The signaling pathway of C_2_H_4_ is controlled by CTR1 (constitutive triple response 1), a serine–threonine protein kinase that acts as a negative regulator, downstream of the receptor and upstream of EIN2. C_2_H_4_ binding results in the inactivation of the receptor–CTR1 complex, and in turn allows activation of a kinase cascade controlling EIN2 and its transcription factors in the nucleus such as EIN3, EIL1, ethylene-responsive element binding proteins (EREBPs)/ethylene-responsive factors (ERFs), which activate the transcription of ethylene-responsive genes (Wang et al., 2002; Liu et al., 2004; Rzewuski and Sauter, 2008; Yoo et al., 2008; Stepanova and Alonso, 2009). EIN2 works downstream of CTR1 and upstream of EIN3 (Alonso et al., 1999). Recently, Qiao et al. (2009) demonstrated that EIN2 protein level is regulated through its degradation by the proteasome in the presence of the hormone via 2 F-Box proteins ETP1 and ETP2; in the presence of C_2_H_4_, ETP1 and ETP2 levels are low, thus increasing EIN2 protein level.

### SEED RESPONSIVENESS TO EXOGENOUS ETHYLENE

The influence of ethylene on seed germination is well documented (Corbineau and Côme, 1995; Kepczynski and Kepczynska, 1997; Matilla, 2000; Matilla and Matilla-Vazquez, 2008). Ethylene, ethephon (an ethylene-releasing compound), or ACC (the precursor of ethylene) stimulate seed germination in numerous species, among which several parasitic plants such as *Orobanche ramosa* (Chun et al., 1979) and some *Striga* species (Egley and Dale, 1970; Bebawi and Eplee, 1986). Application of ethylene promotes germination of either primary dormant or secondary dormant seeds (**Table 1**). It breaks seed coat-imposed dormancy in cocklebur (*Xanthium pennsylvanicum*; Katoh and Esashi, 1975; Esashi et al., 1978), subterranean clover (*Trifolium subterraneum*; Esashi and Leopold, 1969), *Rumex crispus* (Taylorson, 1979) and *Arabidopsis* (Siriwitayawan et al., 2003), and embryo dormancy in apple (*Malus domestica*; Kepczynski et al., 1977; Sinska and Gladon, 1984), sunflower (*Helianthus annuus*; Corbineau et al., 1990), and beechnut (*Fagus sylvatica*; Calvo et al., 2004a). It can also overcome thermodormancy in lettuce (Abeles, 1986) or secondary dormancy in sunflower (Corbineau et al., 1988), *Amaranthus caudatus* (Kepczynski et al., 1996a), and *Amaranthus paniculatus* (Kepczynski and Kepczynska, 1993). Likewise, it stimulates germination of non-dormant seeds placed in non-optimal conditions (Kepczynski and Kepczynska, 1997; Matilla, 2000). For example, it can overcome the inhibition of germination imposed by high temperatures (Abeles, 1986; Gallardo et al., 1991) or osmotic agents (Negm and Smith, 1978; Kepczynski and Karssen, 1985), and alleviates the salinity effect in numerous halophytes (Khan et al., 2009).

**Table 1:** Species whose seed dormancy is broken by ethylene or ethephon, an ethylene-releasing compound, or 1-aminocyclopropane-1-carboxylic acid (ACC).

Species	Type of dormancy	Reference
*Amaranthus caudatus *	Primary and secondary dormancies	Kepczynski and Karssen (1985); Kepczynski et al. (1996a, 2003)
*Amaranthus paniculatus*	Secondary dormancy	Kepczynski and Kepczynska (1993)
*Amaranthus retroflexus*	Primary dormancy	Kepczynski et al. (1996b)
*Arabidopsis thaliana*	Primary dormancy	Siriwitayawan et al. (2003)
*Arachis hypogaea*	Primary dormancy	Ketring and Morgan (1969)
*Chenopodium album*	Primary dormancy	Machabée and Saini (1991)
*Fagus sylvatica*	Embryo primary dormancy	Calvo et al. (2004a)
*Helianthus annuus*	Embryo primary dormancy	Corbineau et al. (1990)
	Secondary dormancy	Corbineau et al. (1988)
*Lactuca sativa*	Thermodormancy	Speer et al. (1974)
**	Secondary dormancy	Abeles (1986)
*Pyrus malus*	Embryo primary dormancy	Kepczynski et al. (1977); Sinska and Gladon (1984)
*Rumex crispus*	Primary and secondary dormancies	Taylorson (1979); Samimy and Khan (1983)
*Stylosanthes humilis*	Primary dormancy	Ribeiro and Barros (2006)
*Trifolium subterraneum*	Primary dormancy	Esashi and Leopold (1969)
*Xanthium pennsylvanicum*	Primary and secondary dormancies	Katoh and Esashi (1975); Esashi et al. (1978)

The stimulatory effect of exogenous ethylene increases with hormone concentration, and the efficient concentrations range from 0.1 to 200 μL L^-1^, depending on species and depth of their dormancy. Ethylene at 1.25 μL L^-1^ allows 100% germination of dormant *Arabidopsis* seeds incubated at 25°C in darkness, when dormant sunflower seeds required 12.5 μL L^-1^ to fully germinate at 15°C. Breaking of dormancy during chilling of apple seeds, or during dry storage of sunflower achenes, results in an increasing sensitivity to ethylene (Sinska, 1989; Corbineau and Côme, 2003). In *Stylosanthes humilis*, non-dormant seeds are at least 50-fold more sensitive to ethylene than freshly harvested dormant ones (Ribeiro and Barros, 2006). Improvement of dormant seed germination does not require a continuous application of ethylene; a short treatment in the presence of this compound is sufficient to improve germination of dormant seeds in various species (Schönbeck and Egley, 1981; Corbineau and Côme, 2003; Kepczynski et al., 2003). Seed responsiveness to ethylene decreases during prolonged pre-incubation under conditions favoring the maintenance of dormancy, probably due to an induction of a secondary dormancy (Speer et al., 1974; Esashi et al., 1978; Jones and Hall, 1984; Corbineau and Côme, 2003).

### INVOLVEMENT OF ETHYLENE BIOSYNTHESIS AND SIGNALING IN SEED GERMINATION

Ethylene production begins as the imbibition phase starts and increases with the germination progression. Its development differs among species (reviewed in Kepczynski and Kepczynska, 1997; Matilla, 2000; Matilla and Matilla-Vazquez, 2008), however, the radicle protrusion through the seed coat is always associated with a peak of ethylene release. A close relationship between the ability to produce ethylene and seed vigor has been reported in numerous species (Samimy and Taylor, 1983; Gorecki et al., 1991; Khan, 1994; Chonowski et al., 1997), and ACC-dependent C_2_H_4_ production was proposed as a marker of seed quality (Corbineau, 2012).

Ethylene production depends on both ACS activity that modulates ACC content, and the activity of ACO, the key enzyme that converts ACC into ethylene. Evolution of ethylene production during germination is associated with an increase in ACO activity, as well as a progressive accumulation of *ACS* and *ACO* transcripts, with generally a sharp increase during endosperm rupture or/and radicle protrusion (Gomez-Jimenez et al., 1998; Matilla and Matilla-Vazquez, 2008; Linkies et al., 2009; Iglesias-Fernandez and Matilla, 2010; Linkies and Leubner-Metzger, 2012). In *Sisymbrium officinale*, *SoACS7* level is very low during seed imbibition, a more notable expression being detected when endosperm rupture reached 50–100%, whereas *SoACO2* expression is detected at early stages during seed imbibition, and then rises during the germination process (Iglesias-Fernandez and Matilla, 2010). Similarly, expression of *PsACO1* in pea (*Pisum sativum*; Petruzzelli et al., 2003) and *BrACO1* in turnip (*Brassica rapa*; Rodriguez-Gacio et al., 2004) is maximal at radicle emergence. In two Brassicaceae species, *Arabidopsis* and *Lepidium sativum*, *ACO1* and *ACO2* have been demonstrated to be the major *ACOs* involved in ethylene synthesis in seeds (Linkies et al., 2009; Linkies and Leubner-Metzger, 2012). In *Lepidium sativum*, the correlation between *ACO1* and *ACO2* transcript accumulation with *in vivo* ACO enzyme activity suggests that *ACO* is regulated at the transcriptional level during germination.

Ethylene has been shown to regulate its own synthesis by inducing *ACO* transcription (Lin et al., 2009). It is required for the stimulation of *ACO* gene expression in pea (Petruzzelli et al., 2000, 2003), beechnut (Calvo et al., 2004b), and turnip (Puga-Hermida et al., 2003). In contrast, expression of *SoACS7* in *Sisymbrium officinale* and *PsACS1* in pea is not affected (Petruzzelli et al., 2000, 2003; Iglesias-Fernandez and Matilla, 2010).

Induction of thermodormancy is often associated with a reduced ethylene production, which may result in chickpea (*Cicer arietinum*) from a greater ACC-malonyltransferase activity and an *S*-AdoMet channeling toward the polyamine pathway, thus reducing ethylene precursor availability (Martinez-Reina et al., 1996), or also from ACO activity inhibition, as observed in chickpea and sunflower (Corbineau et al., 1988; Gallardo et al., 1991). Incubation at high temperature (35°C) of lettuce seeds induces a reduction in ethylene production (Prusinski and Khan, 1990), associated with a complete repression of *LsACS1* and a reduced expression of *ACO-A* (homologous to *AtACO4*; Argyris et al., 2008).

In contrast, treatments (chilling, GA, HCN…) that break seed dormancy often lead to an increase in ethylene production (reviewed in Kepczynski and Kepczynska, 1997; Matilla and Matilla-Vazquez, 2008). Cyanide treatment, which breaks embryo dormancy in apple and sunflower, stimulates ethylene production (Oracz et al., 2008; Gniazdowska et al., 2010). In apple 5-day-old seedlings, it increases ACS and ACO activities (Bogatek et al., 2004), whereas in sunflower it reduces *in vivo* ACC-dependent ethylene production (i.e., *in vivo* ACO activity) and *HaACS* and *HaACO* expression (Oracz et al., 2008). However, in *Arabidopsis*, cold stratification down-regulates the expression of *ACOs*, but results in transient expression of ACS (Narsai et al., 2011; Linkies and Leubner-Metzger, 2012).

Studies using inhibitors of ACS activity (AVG: amino-ethoxyvinylglycine; AOA: amino-oxyacetic acid), ACO activity (CoCl_2_; α-AIB: α-aminoisobutyric acid), or ethylene action (2,5 NBD: 2,5-norbornadiene; STS: silver thiosulfate) demonstrated that ethylene evolved by seeds plays a promotive role in germination and dormancy breakage (Kepczynski et al., 1977, 2003; Sinska and Gladon, 1989; Corbineau et al., 1990; Esashi, 1991; Longan and Stewart, 1992; Gallardo et al., 1994; Hermann et al., 2007). Conversely, application of exogenous ACC stimulates germination of various ethylene-sensitive seeds such as lettuce (Fu and Yang, 1983), sunflower (Corbineau et al., 1990), cocklebur (Satoh et al., 1984), *Amaranthus caudatus* (Kepczynski, 1986) and *Amaranthus retroflexus* (Kepczynski et al., 1996b), chickpea (Gallardo et al., 1994), sugar beet (*Beta vulgaris*; Hermann et al., 2007). Thermodormancy in lettuce, *Amaranthus caudatus* and chickpea is also reversed by exogenous ACC (Gallardo et al., 1996; Kepczynski et al., 2003). This stimulatory effect of ACC suggests that dormancy might be related to low C_2_H_4_ production due to insufficient levels of endogenous ACC, i.e., low ACS activity.

Analysis of mutant lines altered in ethylene biosynthesis and signaling pathway demonstrated the involvement of ethylene in regulating seed germination. Mutations in *ETHYLENE RESISTANT1* (*ETR1*) and *ETHYLENE INSENSITIVE2* (*EIN2*) genes result in poor germination and deeper dormancy compared to wild type, in contrast *constitutive triple response1* (*ctr1*) seeds germinate slightly faster (Bleecker et al., 1988; Leubner-Metzger et al., 1998; Beaudoin et al., 2000; Subbiah and Reddy, 2010). *ERFs* genes might also play a key (pivotal) role in ethylene responsiveness and germination regulation (Leubner-Metzger et al., 1998; Pirrello et al., 2006). In beechnut, Jimenez et al. (2005) demonstrated that the expression of *FsERF1*, a transcription factor involved in C_2_H_4_ signaling and sharing high homology with *Arabidopsis* *ERFs*, increases during dormancy release in the presence of ethephon or after chilling. In sunflower, *ERF1* expression is fivefold higher in non-dormant than in dormant embryos, and also markedly stimulated by gaseous HCN, which breaks dormancy (Oracz et al., 2008). Beechnut *FsERF1* is almost undetectable in dormant seeds incubated under high temperature conditions that maintain dormancy, or in the presence of germination inhibitors, either ABA or AOA, an inhibitor of ethylene biosynthesis, but increases during moist chilling that progressively breaks dormancy (Mortensen et al., 2004; Jimenez et al., 2005). In tomato (*Solanum lycopersicon*), *SlERF2* transcript accumulation is higher in germinating seeds than in non-germinating ones, and its overexpression in transgenic lines results in premature seed germination (Pirrello et al., 2006). Interestingly, in lettuce seeds, expression of genes involved in ethylene signaling (*CTR1*, *EIN2*, and *ETR1*) is less affected by high temperature than that of biosynthesis genes (*ACS* and *ACO*; Argyris et al., 2008).

### CROSSTALK BETWEEN ETHYLENE AND ABA

#### Effect of ABA on ethylene metabolism

The antagonistic interaction between ABA and C_2_H_4_ during germination was demonstrated in numerous species (Leubner-Metzger et al., 1998; Beaudoin et al., 2000; Ghassemian et al., 2000; Kucera et al., 2005; Matilla and Matilla-Vazquez, 2008). In *Arabidopsis* and *Lepidium sativum,* ethylene counteracts the inhibitory effects of ABA on endosperm cap weakening and endosperm rupture (Linkies et al., 2009). ABA also increases the ethylene requirement to release primary and secondary dormancies (Kepczynski and Kepczynska, 1997; Corbineau and Côme, 2003; Kepczynski et al., 2003). Inhibition of germination by ABA is associated with a reduction in ethylene production (Kepczynski and Kepczynska, 1997; Matilla, 2000). ABA clearly inhibits *in vivo* ACO activity, and this inhibition correlates with a decreased accumulation of *ACO* transcripts (Bailly et al., 1992; Petruzzelli et al., 2000, 2003; Linkies et al., 2009). In *Arabidopsis*, the accumulation of *ACO1* transcripts in both the embryo and endosperm during germination is inhibited by ABA, and the high levels of *ACO1* transcripts in ABA-insensitive mutants suggests the regulation of *ACO* expression by ABA (Penfield et al., 2006; Carrera et al., 2008; Linkies et al., 2009). In the embryo, *ACO2* transcript accumulation is also inhibited by ABA (Penfield et al., 2006). In *Lepidium sativum*, inhibition of both *ACO1* and *ACO2* by ABA is restricted to the endosperm cap (Linkies et al., 2009). In accordance, microarray analysis in *Arabidopsis* *aba2* mutant detected an up-regulation of *ACO* transcript accumulation (Cheng et al., 2009). Moreover, inhibition of shoot growth in tomato ABA-deficient mutants, *flacca* and *notabilis*, and in *Arabidopsis* *aba2* results from increased ethylene production (Sharp et al., 2000; LeNoble et al., 2004). In contrast to pea, chickpea, *Lepidium sativum*, and *Arabidopsis*, there is an ABA-mediated up-regulation of ACC accumulation and *ACO* expression in sugar beet seeds (Hermann et al., 2007).

#### Effect of ethylene on ABA metabolism and signaling

Treatment with exogenous ethylene or ACC does not affect ABA content nor expression of genes involved in ABA biosynthesis in *Lepidium sativum* (Linkies et al., 2009) and sugar beet (Hermann et al., 2007). Nevertheless, seeds of *Arabidopsis* ethylene-insensitive mutants, *etr1* and *ein2*, exhibit higher ABA content than wild type and consistently germinate more slowly (Kende et al., 1998; Beaudoin et al., 2000; Ghassemian et al., 2000; Chiwocha et al., 2005; Wang et al., 2007). ABA-GE levels are reduced in *etr1–2* seeds; increased ABA accumulation might therefore be attributed to a decrease in ABA conjugation (Chiwocha et al., 2005). However, ethylene may also regulate other enzymatic steps, since a microarray analysis reported *NCED3* up-regulation in *ein2* and *CYP707A2* down-regulation in *etr1-1* (Cheng et al., 2009). High ABA levels in *ein2* were also associated with an up-regulation of *ABA1* (Wang et al., 2007), which was, however, not detected on microarrays (Cheng et al., 2009).

Several reports suggest that, during germination, ethylene not only acts on ABA metabolism to reduce ABA levels, but also negatively regulates ABA signaling (Gazzarrini and McCourt, 2001; Kucera et al., 2005). Indeed, mutations that reduce ethylene sensitivity (e.g., *etr1*, *ein2*, and *ein6*) result in an increase in ABA sensitivity, while increased ethylene sensitivity in *ctr1* and *eto1* reduces ABA sensitivity (Beaudoin et al., 2000; Ghassemian et al., 2000; Brady and McCourt, 2003; Chiwocha et al., 2005; Kucera et al., 2005; Linkies et al., 2009; Subbiah and Reddy, 2010). Mutations in *CTR1*, for example, enhance the ABA insensitivity of *abi1-1* seeds, when C_2_H_4_-insensitive mutants like *ein2* reduce it (Beaudoin et al., 2000). However, no significant difference in ABA sensitivity is observed in *ein3*, *ein4*, *ein5*, and *ein7* (Subbiah and Reddy, 2010).

In addition, overexpression in *Arabidopsis* seeds of a beechnut tyrosine phosphatase, *FsPTP1*, reduces dormancy, through both ABA signaling down-regulation and *EIN2* up-regulation, suggesting that the negative role of *FsPTP1* in ABA signaling might result from modulation of C_2_H_4_ signaling (Alonso-Ramirez et al., 2011). This central role of EIN2 in mediating cross-links between hormonal response pathways has also been reported in plant response to abiotic and biotic stresses (Wang et al., 2007).

Despite the existence of interactions between the ABA and ethylene signaling pathways, genetic evidence indicates that they may mainly act in parallel, since double mutants obtained by crossing ethylene mutants (*ctr1*, *ein1*, *ein3*, and *ein6*) with the *aba2* mutant exhibit phenotypes resulting from both ABA deficiency and altered ethylene sensitivity (Cheng et al., 2009).

## NITRIC OXIDE HOMEOSTASIS, SIGNALING AND CROSSTALK WITH ABA AND ETHYLENE

### NITRIC OXIDE: CHEMICAL NATURE AND REACTIVITY

Nitric oxide is an inorganic, uncharged, gaseous free radical that can readily diffuse through cell membranes. Upon production, released NO can adjust to the cellular redox environment leading to the formation of diverse biologically active compounds referred to as reactive nitrogen species (RNS; Stamler et al., 1992). Thus, its biological half-life is assumed to be in the order of seconds depending on the redox environment and the initial amount (Saran et al., 1990). While NO production can be beneficial at relatively low levels, uncontrolled accumulation, referred to as nitrosative stress, can result in detrimental consequences in plant cells. A strict control of NO levels is therefore required for cell survival. The regulation of NO biosynthesis, localization, and duration along with the control of NO removal (or storage) is therefore of paramount importance in determining the biological consequences of NO accumulation and thus for its role as secondary messenger (Besson-Bard et al., 2008; Moreau et al., 2010; Baudouin, 2011). The chemical reactivity of NO makes it an unusual signal molecule that can readily act on a wide range of targets, especially proteins (Besson-Bard et al., 2008). The signal it mediates can also be modulated along the signal transduction pathways depending on the biological environment, thus adding to the complexity of NO signaling.

### THE DISTINCT PATHWAYS FOR NITRIC OXIDE BIOSYNTHESIS IN PLANTS AND THEIR RELATIVE CONTRIBUTION IN SEEDS

**FIGURE 4:**
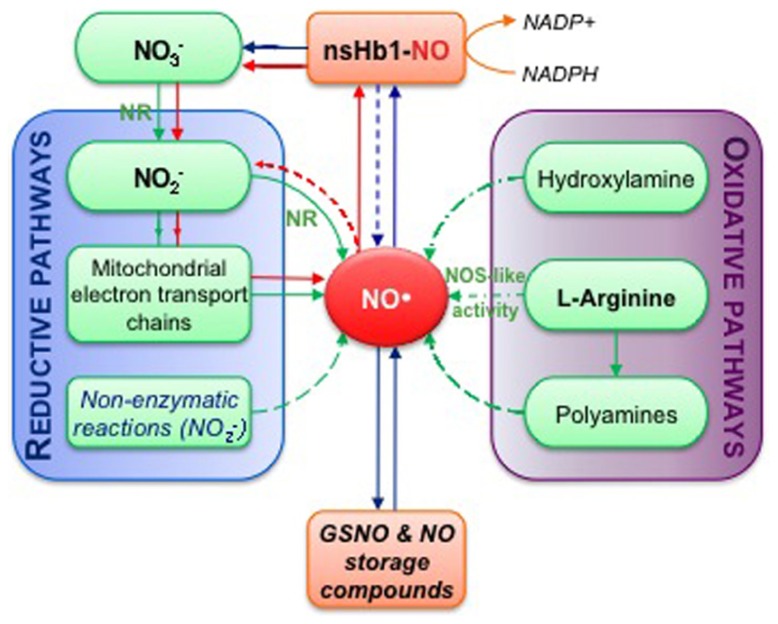
**Simplified overview of NO biosynthesis and homeostasis in plant cells**. This scheme is inspired from Moreau et al. (2010). Nitrate (${\text{NO}}_{3}^{-}$ ) assimilation produces nitrite (${\text{NO}}_{2}^{-}$ ) in a reaction catalyzed by nitrate reductase (NR). The subsequent reduction of nitrite into NO can occur enzymatically, either through NR activity or mitochondrial electron transport chains, and *via* non-enzymatic reactions (reductive pathways). Alternatively, NO synthesis can result from oxidative reactions from hydroxylamine, polyamines or L-arginine (L-Arg; oxidative pathways). NO synthesis from L-Arg could account for the nitric oxide synthase-like (NOS-like) activity detected in plants. The pool of NO is then influenced by non-symbiotic hemoglobin 1 (nsHb1) dioxygenase activity, which converts NO into ${\text{NO}}_{3}^{-}$ . NO can also react with reduced glutathione or thiol groups leading to the reversible formation of *S*-nitrosothiols (e.g., GSNO, *S*-nitrosoglutathione; *S*-nitrosylated proteins). Red arrows highlight the so-called nitrate-NO cycle that may take place under hypoxia. Green arrows correspond to biosynthesis reactions while blue arrows indicate reactions involved in NO homeostasis.

Due to their importance as basis for NO-mediated signaling, the biosynthesis pathways of NO in plants have been the subjects of intense investigations during the last decade (Besson-Bard et al., 2008; Corpas et al., 2009; Moreau et al., 2010; Gupta et al., 2011). The existence of several sources of NO associated with enzymatic or non-enzymatic reactions has been reported but only a few have been completely elucidated so far. Here we will mainly focus on the reactions proven or suggested to be relevant in the context of seed physiology (**Figure 4**), as NO synthesis was previously reviewed in Simontacchi et al. (2007) and Sirova et al. (2011).

#### Nitric oxide synthase-like activity

In animals, NO biosynthesis is mainly catalyzed by three isoforms of NO synthase (NOS; Alderton et al., 2001). These enzymes metabolize L-arginine (L-Arg) into L-citrulline and NO *via* the following reaction:

$$\text{L-Arg}+\text{NAD(P)H,}{\text{H}}^{+}+{\text{O}}_{2}\Rightarrow \text{L}-\text{citrulline}+\text{NAD}{\left(\text{P}\right)}^{+}+{\text{H}}_{2}\text{O}+\text{NO}$$

To date, despite the identification of a green alga NOS (Foresi et al., 2010), the search for a NOS homolog enzyme in higher plants only encountered failure, although biochemical assay highlighted the existence of a NOS-like activity in several plant tissues and organelles (Fröhlich and Durner, 2011). Moreover, exogenous application of NOS inhibitors (structural analogs of L-Arg such as L-NAME, *N*-nitro-L-arginine methyl ester) significantly reduced NO release under diverse conditions in several plant species (Crawford, 2006). Using these approaches, a NOS-like activity was detected in sorghum (*Sorghum bicolor*) and soybean (*Glycine max*) imbibed seeds (Simontacchi et al., 2007). Nonetheless, the liability of such proofs is now debated in light of the discovery of other L-Arg-dependent NO synthesis pathways (Tun et al., 2006). Moreover, the recent finding that L-NAME can affect NO production by interfering with nitrate reductase (NR) activity discredits its use as a NOS inhibitor in plants (Rasul et al., 2012). Thus, after more than a decade of intense research in the area and despite the proven occurrence of L-Arg-dependent NO biosynthesis, the mere existence of NOS is now questioned in plants (Fröhlich and Durner, 2011).

#### Nitrate reductase

Apart from its well-known role in nitrate reduction and assimilation, the cytosolic NR has been shown to catalyze the reduction of nitrite to NO, using NAD(P)H as electron donor, both *in vitro* and *in vivo*, *via* the following reaction (Yamasaki et al., 1999; Rockel et al., 2002):

$$\text{NAD(P)H}+{3\text{H}}_{3}{\text{O}}^{+}+{2\text{NO}}_{2}^{-}\Rightarrow \text{NAD}{\text{(P)}}^{+}+\text{2NO}+{5\text{H}}_{2}\text{O}$$

*In vivo*, NR would be responsible at least in part for the basal level of NO production with a low reduction efficiency (in the order of 1% of the total NR activity). However, the nitrite reductase activity of NR (NR-NiR) can drastically increase under certain conditions such as oxygen deprivation (Rockel et al., 2002). Overall, conditions leading to NR-mediated nitrite production exceeding the rate of nitrite removal can lead to a substantial increase in NO production by NR. Both the nitrate and nitrite reductase activities of NR are tightly controlled by post-translational modifications (PTM; Lillo et al., 2004; Park et al., 2011; Wang et al., 2011). In *Arabidopsis*, NR and NR-NiR activities are stimulated by sumoylation mediated by the E3 SUMO ligase AtSIZ1 (Park et al., 2011). Furthermore, the H_2_O_2_-induction of NO biosynthesis in *Arabidopsis* roots was recently proposed to depend on mitogen-activated protein kinase 6 (MPK6)-mediated phosphorylation of one of the NR isoforms (Ser 627 in *Arabidopsis* NIA2; Wang et al., 2010, 2011). Moreover, NO was reported to inhibit NR activity in wheat leaves (Rosales et al., 2011). In *Arabidopsis* seedlings, GA may also negatively regulate light-induced NR activity at post-translational level (Zhang et al., 2011).

Distinct studies reported an implication of NR in NO-mediated signal transduction pathways (Bright et al., 2006; Neill et al., 2008; Gupta et al., 2011). In seeds, the NO-mediated positive effect of ${\text{NO}}_{2}^{-}$ and ${\text{NO}}_{3}^{-}$ on dormancy release supports an involvement of nitrite-dependent reductive pathways in NO biosynthesis, possibly via NR-NiR activity or at least depending on NR activity in the case of exogenous ${\text{NO}}_{3}^{-}$ (Bethke et al., 2006b). Accordingly, NR activity was detected concomitantly with a NOS-like activity in soybean and sorghum embryonic axes, both enzymatic activities appeared to parallel the accumulation of NO upon seed imbibition (Simontacchi et al., 2007).

In *Arabidopsis*, NR is encoded by two homologous genes, *NIA1* and *NIA2* (Wilkinson and Crawford, 1991). The relative contribution of these two isoforms to NO production was suggested to differ with a possible predominant involvement of NIA1 in NO production (Baudouin, 2011). Despite NO has been demonstrated to break seed dormancy (Bethke et al., 2006b; Liu et al., 2009), NR involvement in *Arabidopsis* seed germination remains unclear. Two distinct research groups assessed the germination characteristics of the *nia1nia2* double mutant (also named G′4-3), obtained by Wilkinson and Crawford (1993). In the first study, G′4-3 seeds were found to be less dormant than wild type seeds (Alboresi et al., 2005), but more dormant in the second (Lozano-Juste and Leon, 2010). Differences in culture environments of mother plants, germination conditions or duration of seed storage may explain these contrasted results (Clerkx et al., 2004; Matakiadis et al., 2009).

#### Polyamines and hydroxylamines

Upon exogenous application of the polyamines, spermine (spm) and spermidine (spd), a rapid NO production from *Arabidopsis* seedlings has been observed under aerobic conditions (Tun et al., 2006). In plants, the tri-amine Spd and tetra-amine Spm are formed by successive additions of aminopropyl groups [resulting from *S*-AdoMet decarboxylation] to the diamine putrescine (Put; reviewed in Wimalasekera et al., 2011a). Put can be synthesized either from L-Arg (by L-Arg decarboxylase) or from L-ornithine (by ornithine decarboxylase). However, as *Arabidopsis* lacks ornithine decarboxylase activity, polyamines are exclusively produced from L-Arg (Hanfrey et al., 2001). Thus, NO biosynthesis from polyamines can be considered as a L-Arg-dependent pathway in *Arabidopsis*.

Plant cells are also able to produce NO through hydroxylamine oxidation and this reaction is promoted by reactive oxygen species (ROS) accumulation (Rümer et al., 2009). Thus, NO might be responsible for the positive effect of exogenous hydroxylamines on seed germination (Hendricks and Taylorson, 1974). However, the relevance of such pathway to NO synthesis remains unclear.

#### Nitric oxide production in the apoplast

The existence of a root specific plasma membrane nitrite-NO reductase (Ni-NOR) was reported in tobacco (*Nicotiana tabacum*; Stöhr et al., 2001). This enzyme would catalyze the reduction of nitrite into NO in the apoplast and could act in tandem with a plasma membrane-bound NR (PM-NR; Eick and Stöhr, 2012). Its implication has been proposed in several physiological processes in roots (Stöhr and Stremlau, 2006), but has not been so far investigated in seeds.

The non-enzymatic reduction of nitrite to NO can also occur under acidic pH and could be promoted by the presence of reductants (Mallick et al., 2000):

$${2\text{HNO}}_{2}\Rightarrow \text{NO}+{\text{NO}}_{2}+{\text{H}}_{2}\text{O}\Rightarrow 2\text{NO}+{2\text{O}}_{2}+{\text{H}}_{2}\text{O}$$

This non-enzymatic reaction may be of paramount importance in seeds as an intense NO production was observed during early *Arabidopsis* seed imbibition next to the aleurone layer (Liu et al., 2009). Sodium nitroprusside (SNP) releases dormancy by generating both NO and cyanide. In C24 dormant seeds, the cell impermeable NO scavenger, cPTIO (2-(4-carboxyphenyl)phenyl-4,4,5,5-tetramethylimidazoline-1-oxyl 3-oxide), was demonstrated to efficiently impede SNP dormancy release, suggesting that the apoplast might be either an important pathway for NO movement or a site for NO production (Bethke et al., 2006b).

#### Mitochondrial respiration

Depending on the oxygen availability, several hemeproteins can either act as NO scavengers or NO producers. In hypoxic mitochondria, deoxyhemeproteins can catalyze a NR-independent nitrite reduction into NO using electrons from the electron transport chain (Planchet et al., 2005). The re-oxidation of NO into nitrite can then occur either non-enzymatically inside the mitochondria, or in the cytosol, through the nicotinamide adenine dinucleotide phosphate (NADPH)-dependent dioxygenase activity of class-1 non-symbiotic hemoglobin (nsHb1) that metabolizes NO into nitrate, which is subsequently reduced into nitrite by NR (Igamberdiev and Hill, 2004; Perazzolli et al., 2004). These reactions constitute the so-called hemoglobin-NO cycle (displayed in red in **Figure 4**; Igamberdiev et al., 2010). nsHb1 proteins participate in NO scavenging, thereby playing an essential role in NO homeostasis. Accordingly, modulation of nsHb1 expression in plants was shown to directly impact NO levels at distinct developmental stages including in seeds (Hebelstrup and Jensen, 2008; Thiel et al., 2011) and in diverse environmental conditions (Dordas, 2009; Cantrel et al., 2011).

The very active mitochondrial respiration upon seed imbibition may result in an oxygen consumption exceeding the atmospheric diffusion, thus leading to localized hypoxia in germinating seeds (Benamar et al., 2008). In such conditions, nitrite-dependent NO production may occur in mitochondria and modulate respiration through reversible NO-mediated inhibition of cytochrome *c* oxidase (COX), thereby regulating oxygen consumption to avoid anoxia (Benamar et al., 2008). Therefore, this nitrite-dependent NO biosynthesis in mitochondria may be of significant importance in germinating seeds. However, its possible role in NO-mediated dormancy release has not yet been established.

Overall, current evidence supports the co-existence of several distinct NO biosynthesis pathways in seeds. Their relative contribution is probably highly dependent on both oxygen and ROS levels that may change along the time-course of imbibition. Further investigations will be required to elucidate the regulation of NO accumulation during seed imbibition.

#### S-nitrosoglutathione: a reversible “storage” pool of nitric oxide?

As for plant hormones, any mechanism directly influencing NO levels besides biosynthesis pathways may have a pivotal role in the regulation of NO signaling. In particular, since NO can react with reduced glutathione (GSH) to form *S*-nitrosoglutathione (GSNO), GSNO has been proposed to constitute a storage and transport form for NO in plants and seeds (Sakamoto et al., 2002). Such modulation of NO storage pool would have a significant impact on NO levels. GSNO can further be metabolized by the GSNO reductase (GSNOR). Accordingly, *gsnor* mutants have multiple phenotypes suggesting GSNOR involvement in several growth and developmental processes including seed germination (Lee et al., 2008; Holzmeister et al., 2011; Kwon et al., 2012).

### MOLECULAR TARGETS OF NITRIC OXIDE IN SEEDS

Due to its chemical nature, NO is highly reactive and can interact with diverse molecules in plant cells. A number of NO-regulated genes have been identified in plants (Besson-Bard et al., 2009). These genes encode proteins involved in a wide range of functions from signal transduction to stress responses. However, the main challenge remains to pinpoint the direct molecular targets of NO, which are still poorly documented in plants. However, it is generally assumed that proteins constitute direct relevant NO targets. Besides its capacity to bind to transition metals of metalloproteins, NO can cause protein PTM, such as cysteine *S*-nitrosylation or tyrosine nitration (Moreau et al., 2010). These modifications remain poorly characterized in plants and particularly in seeds. However, as discussed below, there is strong experimental evidence indicating that NO signaling in seeds could principally rely on PTM of specific proteins (Delledonne, 2005).

Many *S*-nitrosylated proteins identified in plants are implicated in various metabolic processes (Lindermayr et al., 2005; Abat et al., 2008; Romero-Puertas et al., 2008; Tanou et al., 2009; Palmieri et al., 2010). In dry *Arabidopsis* seeds, a β-subunit of the mitochondrial ATP synthase complex was found to be *S*-nitrosylated, suggesting that NO could participate in the regulation of the seed energy status (Arc et al., 2011). In wheat seeds, a parallel increase in NO and protein *S*-nitrosylation was reported during *sensu stricto* germination (Sen, 2010). At least 13 modified proteins were detected, but not identified. In recalcitrant *Antiaris toxicaria* seeds, desiccation impedes subsequent germination by enhancing H_2_O_2_ accumulation (Bai et al., 2011). This stress is associated with an increased carbonylation and a reduced *S*-nitrosylation of the antioxidant enzymes of the ascorbate-GSH pathway. Conversely, NO pre-treatments promote germination of desiccated seeds through PTM pattern reversion that enhances antioxidant enzyme activities (Bai et al., 2011). The balance between carbonylation and *S*-nitrosylation of these proteins was proposed to act as molecular switch tuning their activity according to the redox environment (Lounifi et al., 2013).

### CROSSTALK BETWEEN NO, ETHYLENE, AND ABA

In stomatal guard cells, ABA-induced stomatal closure is mediated by the successive accumulation of ROS and NO, acting as secondary messengers in ABA signaling (Neill et al., 2008). Even though similar actors are present in seeds, the picture is quite different, as both ROS and NO counteract ABA-inhibition of seed dormancy release and germination (Bethke et al., 2006b; Liu et al., 2010). This obvious discrepancy of NO action between seeds and stomata highlights the specificity of the seed signaling pathways (**Figure 2**).

In imbibed seeds, the application of ABA biosynthesis inhibitors, fluridone or norflurazon, reduces ABA neo-synthesis and promotes dormancy release and germination. In tomato seeds, the NO scavenger, cPTIO, was shown to prevent germination stimulation by fluridone (Piterkova et al., 2012). Conversely, in dormant *Arabidopsis* C24 seeds, SNP enhances the positive effect of norflurazon on germination and also decreases seed sensitivity to exogenous ABA (Bethke et al., 2006a). Taken together, these results suggest that NO reduces both ABA accumulation and sensitivity. In agreement, pharmacological experiments demonstrated that NO enhances *CYP707A2* gene expression in *Arabidopsis* seeds (Liu et al., 2009). Indeed, during the first stage of seed imbibition, a rapid accumulation of NO, possibly at the endosperm layer, was suggested as required for rapid ABA catabolism and dormancy breaking. A similar NO accumulation during imbibition was also observed in germinating seeds from other species (Simontacchi et al., 2007). Recently, in *Arabidopsis*, NO was suggested to act upstream of GA in a signaling pathway leading to vacuolation of protein storage vacuoles in aleurone cells, a process inhibited by ABA (Bethke et al., 2007a). Since the growth of isolated embryos was unaffected by NO donors or scavengers, the endosperm layer might be the primary site of NO synthesis and action in seeds, and in accordance was shown to perceive and respond to NO (Bethke et al., 2007a). Besides its effect on the hormonal balance, it has been speculated that NO may accelerate flux through the pentose phosphate pathway by indirectly increasing the oxidation of NADPH (Hendricks and Taylorson, 1974; Bethke et al., 2007b). An increase in glucose catabolism via this pathway may in turn promote dormancy release (Roberts and Smith, 1977).

Several lines of evidence suggest that NO crosstalk with ABA and ethylene may involve protein modifications. Among the proteins recently identified as candidates for a regulation by tyrosine nitration in *Arabidopsis* seedlings (Lozano-Juste et al., 2011), at least two may be involved in the interplay between ABA and NO in seeds. The first one is the Moco sulfurase ABA3 that catalyzes the conversion from the de-sulfo to the sulfo form of the Moco (Wollers et al., 2008). The de-sulfo form of Moco (also call the “oxo” form) is the co-factor of NR, involved in nitrite and NO generation in plants while the sulfo form is the co-factor of the aldehyde oxydase required for the last step of ABA synthesis (Mendel, 2007). If proven, modulation of ABA3 activity by nitration could affect the equilibrium between ABA and NO production in plants. The second protein is the E3 SUMO ligase AtSIZ1 recently demonstrated to stimulate NR and NR-NiR activities, and negatively regulate ABA signaling by ABI5 sumoylation (Miura et al., 2009; Park et al., 2011). Thus, such modifications could have an important impact in seeds. Similarly, PTM contribution in the NO regulation of ethylene action has been also reported. In *Arabidopsis*, the up-accumulation of NO under hypoxia stimulates ethylene biosynthesis, possibly through PTM of key enzymes such as ACS and ACO by *S*-nitrosylation (Hebelstrup et al., 2012). In contrast, ethylene biosynthesis can be reversibly inhibited by NO through *S*-nitrosylation of methionine adenosyltransferase (MAT), leading to the reduction of the *S*-AdoMet pool (Lindermayr et al., 2006).

*S*-AdoMet is the precursor of ethylene and polyamines, thus a negative feedback regulation may exist between ethylene and the polyamine-dependent NO biosynthesis. Consistently, NO and ethylene accumulation are negatively correlated in ripe fruits (Manjunatha et al., 2012). In addition, exogenous Spm was shown to reduce ethylene production in apple seeds (Sinska and Lewandowska, 1991). Accordingly, an antagonism may exist between a positive polyamine effect mediated by NO and a negative effect due to a competition with ethylene biosynthesis for *S*-AdoMet. Furthermore, a copper amine oxidase (CuAO1) involved in polyamine catabolism has also been shown to regulate NO biosynthesis and participate to ABA signaling (Wimalasekera et al., 2011b). Indeed, seedlings of *Arabidopsis* *cuao1* mutant are impaired in both polyamine and ABA-induced NO synthesis, and mutant seeds also display a reduced sensitivity to exogenous ABA during germination (Wimalasekera et al., 2011b).

As mentioned above, in Brassicaceae species, ethylene positively regulates seed germination by stimulating the weakening and rupture of seed testa and endosperm by counteracting the inhibitory action of ABA on radicle protrusion (Linkies et al., 2009). In apple embryos, inhibition of ethylene biosynthesis prevents the promotion of dormancy release and germination by NO donors (Gniazdowska et al., 2007). Dormancy breaking of apple seeds by NO induces a transient production of ROS, stimulating ethylene accumulation thanks to an increase in both ACS and ACO activity (Gniazdowska et al., 2010). NO may also act on ethylene signaling since EREBPs were described as a class of transcription factors induced by NO (Parani et al., 2004). Moreover during tobacco seed germination, EREBP-3 that is transiently induced just before endosperm rupture is stimulated by ethylene and inhibited by ABA (Leubner-Metzger et al., 1998). Therefore, a synergic link seems to exist, at different levels, between NO and ethylene during seed germination, that counteracts ABA action.

## CONCLUSION

Significant advances have been recently obtained in the understanding of the ABA and ethylene metabolism and signaling pathways. In contrast, current knowledge on NO biosynthesis, signaling and action is far too incomplete, especially in seeds, and would require further investigation. Future research efforts should also lead to the identification of downstream target genes of signaling components, in order to fully understand how ABA is able to induce and maintain dormancy, or ethylene to stimulate germination. Moreover unraveling the role of post-translational mechanisms will be particularly crucial to developing a deeper understanding of hormonal pathways and deciphering NO regulatory network.

Nitric oxide and ethylene crosstalk with ABA involves interactions at multiple levels in metabolism and signaling pathways. It would be important to discriminate the hierarchy among these signaling pathways, identify major regulatory nodes and determine whether the environmental factors, which regulate germination and dormancy, modulate this hierarchy.

Moreover, control of seed dormancy and germination involves distinct physiological processes, in tissues of different origin, to achieve a coordinated regulation of embryo arrest or growth and surrounding structure maintenance or rupture. Although hormonal signaling networks in seeds and whole plants share common components, sets of specific regulatory factors, among which only few are known, are likely working in restricted seed territories. Current research combining genetic tools and recent technologies including microdissection, transcriptome profiling, high-throughput proteomics, metabolomics, and system biology, should help to identify missing regulatory components and unravel complex interactions between signal transduction pathways.

## Conflict of Interest Statement

The authors declare that the research was conducted in the absence of any commercial or financial relationships that could be construed as a potential conflict of interest.
